# Effect of intraoperative personalized goal-directed hemodynamic management on acute myocardial injury in high-risk patients having major abdominal surgery: a post-hoc secondary analysis of a randomized clinical trial

**DOI:** 10.1007/s10877-022-00826-0

**Published:** 2022-02-24

**Authors:** Karim Kouz, Alina Bergholz, Oliver Diener, Maximilian Leistenschneider, Christina Thompson, Friederike Pichotka, Constantin Trepte, Edzard Schwedhelm, Thomas Renné, Linda Krause, Julia Y. Nicklas, Bernd Saugel

**Affiliations:** 1 Department of Anesthesiology, Center of Anesthesiology and Intensive Care Medicine, University Medical Center Hamburg-Eppendorf, Martinistrasse 52, 20246 Hamburg, Germany; 2 Institute of Clinical Chemistry and Laboratory Medicine, University Medical Center Hamburg-Eppendorf, Hamburg, Germany; 3 Institute of Clinical Pharmacology and Toxicology, University Medical Center Hamburg-Eppendorf, Hamburg, Germany; 4 Center for Thrombosis and Hemostasis (CTH), Johannes Gutenberg University Medical Center, Mainz, Germany; 5 Irish Centre for Vascular Biology, School of Pharmacy and Biomolecular Sciences, Royal College of Surgeons in Ireland, Dublin, Ireland; 6 Department of Medical Biometry and Epidemiology, University Medical Center Hamburg-Eppendorf, Hamburg, Germany; 7 Outcomes Research Consortium, Cleveland, OH USA

**Keywords:** Cardiac output, Cardiovascular dynamics, Goal-directed therapy, Hemodynamic monitoring, Myocardial injury after noncardiac surgery, Randomized trial

## Abstract

Acute myocardial injury is common after noncardiac surgery and associated with mortality. Impaired intraoperative cardiovascular dynamics are a risk factor for acute myocardial injury. Optimizing intraoperative cardiovascular dynamics may thus reduce acute myocardial injury. We aimed to investigate the effect of intraoperative personalized goal-directed hemodynamic management on the incidence of acute myocardial injury. We hypothesized that personalized goal-directed hemodynamic management reduces the incidence of acute myocardial injury compared to routine hemodynamic management in high-risk patients having major abdominal surgery. We performed a post-hoc secondary analysis of a randomized clinical trial including 180 high-risk major abdominal surgery patients that were randomized to personalized goal-directed hemodynamic management or routine hemodynamic management. We compared the incidences of acute myocardial injury—defined according to the Fourth Universal Definition of Myocardial Infarction (2018)—between patients randomized to personalized goal-directed hemodynamic management or routine hemodynamic management by calculating the relative and absolute risk reduction together with 95% Wald confidence intervals and P values. Acute myocardial injury occurred in 4 of 90 patients (4%) in the personalized goal-directed hemodynamic management group and in 12 of 90 patients (13%) in the routine hemodynamic management group (relative risk: 0.33, 95% confidence interval: 0.11 to 0.99, P = 0.036; absolute risk reduction: − 9%, 95% confidence interval: − 17% to − 0.68%, P = 0.034). In this post-hoc secondary analysis, intraoperative personalized goal-directed hemodynamic management reduced the incidence of acute myocardial injury compared to routine hemodynamic management in high-risk patients having major abdominal surgery. This needs to be confirmed in larger prospective trials.

**Supplementary Information:**

The online version contains supplementary material available at 10.1007/s10877-022-00826-0.

## Introduction

Acute myocardial injury is common in patients having noncardiac surgery and associated with postoperative mortality [1–5]. Acute myocardial injury is defined by a cardiac troponin elevation [6, 7]. Besides cardiac troponins, N-terminal pro-brain natriuretic peptide (NT-proBNP) is also released in response to acute myocardial injury [8]—and also associated with postoperative morbidity and mortality [9].

Intraoperative hypotension—reflecting impaired intraoperative cardiovascular dynamics [10]—is a modifiable risk factor for acute myocardial injury in patients having noncardiac surgery [11, 12]. One may thus assume that optimizing cardiovascular dynamics during surgery reduces the incidence of acute myocardial injury. Intraoperative cardiovascular dynamics can be optimized by goal-directed hemodynamic management that helps avoiding hypotension and low blood flow states. However, the effect of intraoperative goal-directed hemodynamic management on acute myocardial injury remains scarcely investigated.

In a recent randomized controlled clinical trial, we showed that intraoperative personalized goal-directed hemodynamic management reduces the incidence of postoperative clinical complications compared to routine hemodynamic management in high-risk patients having major abdominal surgery [13]. However, in the original trial, we did not systematically investigate acute myocardial injury by perioperative biomarker screening [13].

We thus now conducted a post-hoc secondary analysis of the original trial [13] to investigate the effect of intraoperative personalized goal-directed hemodynamic management on the incidence of acute myocardial injury. We hypothesized that personalized goal-directed hemodynamic management reduces the incidence of acute myocardial injury compared to routine hemodynamic management in high-risk patients having major abdominal surgery. Additionally, we investigated the effect of personalized goal-directed hemodynamic management on perioperative troponin I and NT-proBNP changes.

## Methods

### Ethics

The original trial and the present study were approved by the ethics committee (Ethikkomission der Ärztekammer Hamburg, Hamburg, Germany; chair: Prof. Dr. Rolf Stahl, registration number PV5018) on 4 August 2015 and 2 December 2020 and all patients provided written informed consent.

### Study design and setting

We performed a post-hoc secondary analysis of a randomized clinical trial [13] that was conducted between May 2016 and June 2017 at the University Medical Center Hamburg-Eppendorf, Hamburg, Germany. The original trial was registered at ClinicalTrials.gov (NCT02834377) in May 2016. The statistical analysis plan was approved by the authors before analyses began but was not publicly available. This manuscript adheres to the applicable Strengthening the Reporting of Observational Studies in Epidemiology (STROBE) Statement [14].

### Patients and protocol of the original trial

Adult high-risk patients scheduled for major abdominal surgery expected to last at least 90 min or cause blood loss exceeding 1000 ml were enrolled into the original trial [13]. Patients who were pregnant, had palliative or emergency surgery, or participated in another trial were excluded [13].

Patients were randomized to intraoperative personalized goal-directed hemodynamic management (targeting baseline cardiac index measured non-invasively one day before surgery) or to routine hemodynamic management [13]. In patients randomized to personalized goal-directed hemodynamic management, cardiac index was measured using pulse wave analysis during surgery and baseline cardiac index was targeted using a predefined treatment algorithm including fluid challenges and, if necessary, dobutamine [13]. Mean arterial pressure was maintained between 65 and 90 mmHg. Patients randomized to routine hemodynamic management were treated as per anesthesiologist preference—with cardiac index monitoring being available on request. Mean arterial blood pressure was maintained above 65 mmHg.

### Measurement of troponin I and N-terminal pro-brain natriuretic peptide

Blood samples were collected before the induction of general anesthesia and three days after surgery. After centrifugation, serum aliquots were separated into Eppendorf tubes and stored at − 80 °C until transfer to our central laboratory for batched analysis. Serum troponin I was measured using the Siemens Atellica IM High-Sensitivity Troponin I Assay (Siemens Healthineers, Erlangen, Germany). The 99th percentile of a reference population for this assay is 38.6 ng/l for women and 53.5 ng/l for men [15]. Serum NT-proBNP was measured using the Siemens Atellica IM NT-proBNP assay (Siemens Healthineers). Serum troponin I and serum NT-proBNP were measured at the Institute of Clinical Chemistry and Laboratory Medicine at the University Medical Center Hamburg-Eppendorf.

### Myocardial injury endpoints

We defined acute myocardial injury according to the definition of “myocardial injury and infarction associated with non-cardiac procedures” set forth in the Fourth Universal Definition of Myocardial Infarction (2018) [7] as a postoperative troponin I concentration above the sex-specific 99th percentile upper reference limit with (1) a ≥ 60% increase from baseline when baseline troponin I concentration was below the sex-specific 99th percentile upper reference limit, or (2) a ≥ 20% increase from baseline when baseline troponin I concentration was above the sex-specific 99th percentile upper reference limit. Using this definition is recommended by an expert consensus panel of the “Standardized Endpoints in Perioperative Medicine (StEP)” initiative [6]. We also investigated relative changes in postoperative troponin I and NT-proBNP concentrations compared to baseline troponin I and NT-proBNP concentrations, i.e., the postoperative minus the preoperative concentration divided by the preoperative concentration.

### Statistical analysis

Descriptive results are presented as medians with 25th percentiles and 75th percentiles for continuous data and as absolute frequencies and percentages for categorical data.

Incidences of acute myocardial injury in the personalized goal-directed hemodynamic management and the routine hemodynamic management group are illustrated using stacked bar charts. We compared the incidences of acute myocardial injury between patients randomized to personalized goal-directed hemodynamic management or routine hemodynamic management by calculating the relative risk (i.e., risk ratio) and the absolute risk reduction together with 95% Wald confidence intervals (CI) and P values (Chi-squared test).

To illustrate preoperative and postoperative troponin I and NT-proBNP concentrations in patients randomized to personalized goal-directed hemodynamic management and routine hemodynamic management we computed spaghetti plots and violin plots with overlaying boxplots. We compared relative changes in troponin I and NT-proBNP concentrations in patients randomized to personalized goal-directed hemodynamic management and routine hemodynamic management using the Wilcoxon rank-sum test with continuity correction.

We used R version 3.5.3 (R Foundation for Statistical Computing, Vienna, Austria) for statistical analyses.

## Results

We excluded 8 of the 188 patients included in the original trial because blood samples were missing. Therefore, we included 180 patients (90 patients in the personalized goal-directed hemodynamic management group and 90 patients in the routine hemodynamic management group) in this post-hoc secondary analysis (Table 1).

**Table 1:** Baseline characteristics

Characteristic	Personalized goal-directed hemodynamic management group (n = 90)	Routine hemodynamic management group (n = 90)
Age, years	66 (56–75)	63 (55–74)
Male, n (%)	52 (58)	57 (63)
Height, cm	174 (168–180)	174 (167–178)
Weight, kg	76 (61–90)	75 (65–85)
**Abdominal surgery procedure category**		
General surgery, n (%)	56 (62)	54 (60)
Urological surgery, n (%)	5 (6)	16 (18)
Gynecological surgery, n (%)	16 (18)	8 (9)
Aortic surgery, n (%)	13 (14)	12 (13)
**American Society of Anesthesiologists physical status**		
II, n (%)	10 (11)	13 (14)
III, n (%)	67 (74)	66 (73)
IV, n (%)	13 (14)	10 (11)
**Clinical characteristics**		
Duration of surgery, min	213 (159–290)	260 (180–321)
Crystalloids, n (%)	90 (100)	90 (100)
Crystalloids, ml	2730 (2000–3613)	3000 (2000–4063)
Colloids, n (%)	42 (47)	49 (54)
Colloids, ml	1000 (500–1500)	1000 (500–1500)
Packed red blood cells, n (%)	24 (27)	29 (32)
Packed red blood cells, units	3 (2–4)	3 (2–5)
Fresh frozen plasma, n (%)	4 (4)	16 (18)
Fresh frozen plasma, units	7 (3–40)	6 (4–8)
Norepinephrine, n (%)	89 (99)	90 (100)
Norepinephrine dose, µg kg^−1^ min^−1^	0.11 (0.06–0.19)	0.13 (0.09–0.20)
Dobutamine, n (%)	34 (38)	8 (9)
Dobutamine dose, µg kg^−1^ min^−1^	2.0 (1.2–2.9)	1.6 (0.7–2.3)

Categorial variables are presented as number and percentage, continuous variables as median with 25th percentile and 75th percentile

**Fig. 1:**
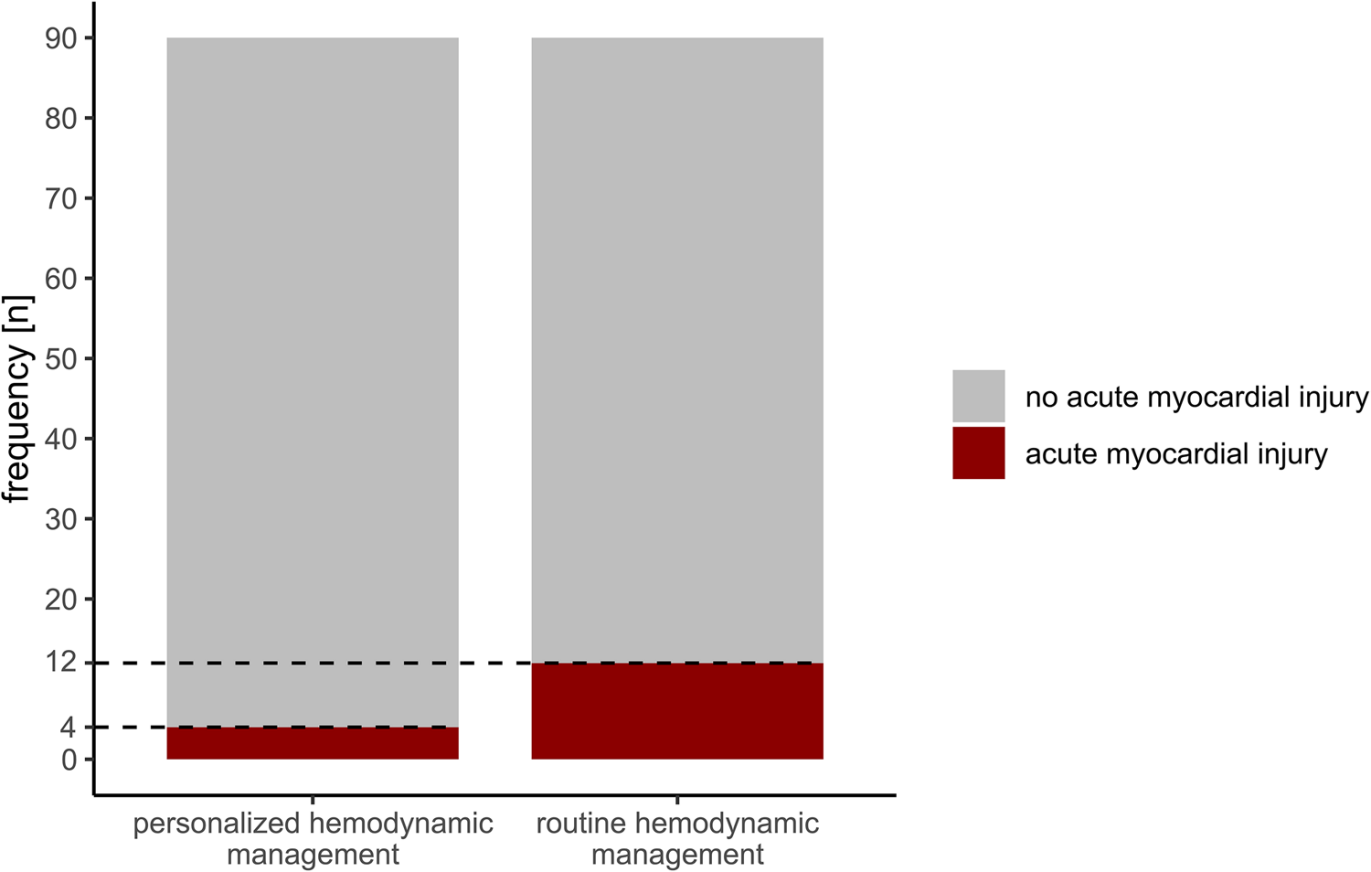
Stacked bar chart showing the number of patients with (red) and without (grey) acute myocardial injury in the personalized goal-directed hemodynamic management group and the routine hemodynamic management group

**Fig. 2:**
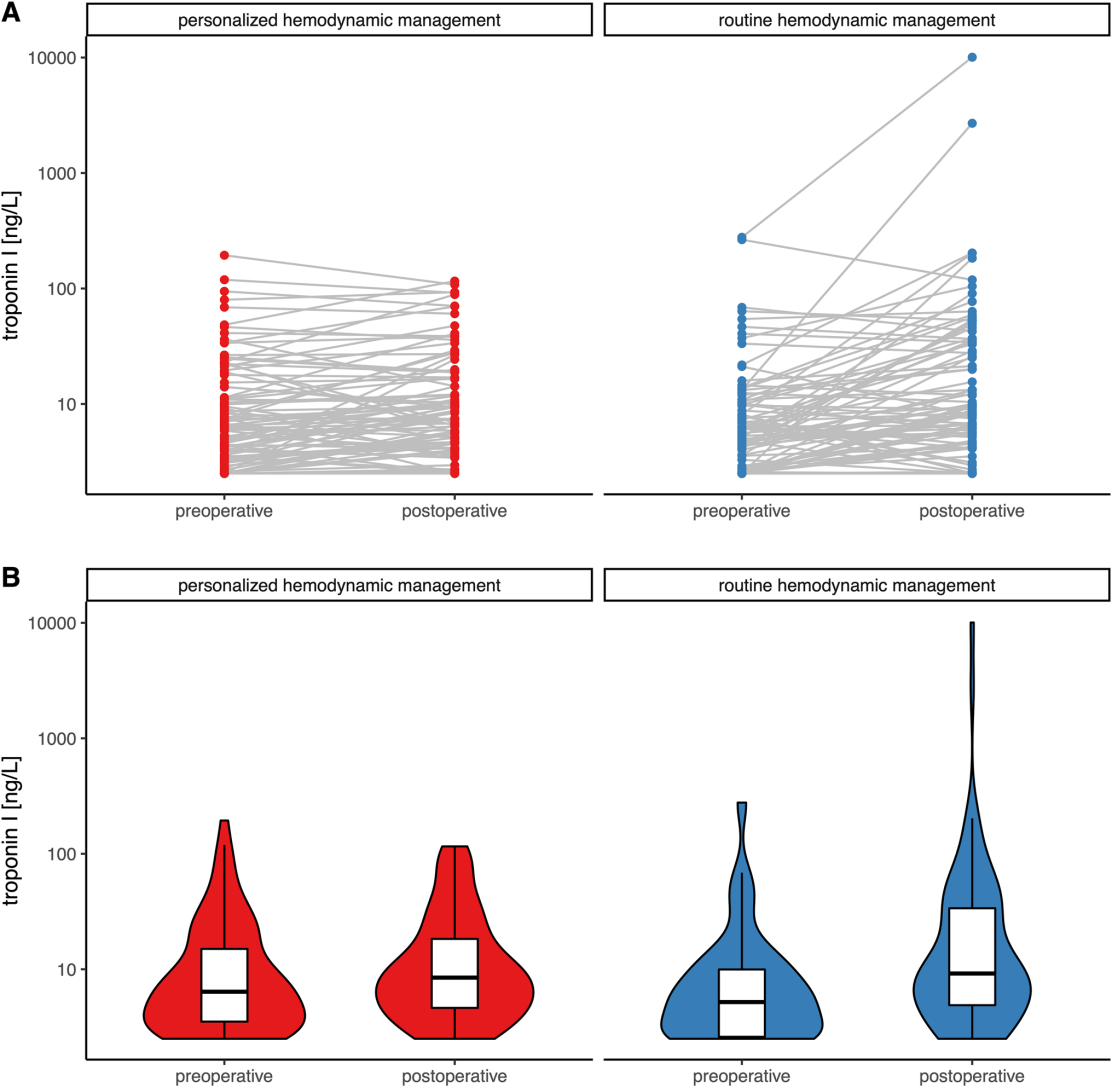
Spaghetti plots (**A**) and violin plots with overlaying boxplots (**B**) showing preoperative and postoperative troponin I concentrations in patients randomized to personalized goal-directed hemodynamic management and routine hemodynamic management. Violin plots represent densities estimated by a Gaussian kernel using automated bandwidth selection with a constant area of all violins and trimming to the data range. In the boxplots, boxes represent the 25th and 75th percentile and the range between them is the interquartile range. Inside the boxes, bold horizontal lines represent medians. The whiskers (extensions from the box) indicate the lowest and highest value no further than 1.5 times the interquartile range

Acute myocardial injury occurred in 4 of 90 patients (4%) in the personalized goal-directed hemodynamic management group and in 12 of 90 patients (13%) in the routine hemodynamic management group (relative risk: 0.33, 95% CI: 0.11 to 0.99, P = 0.036; absolute risk reduction: − 9%, 95% CI: − 17% to − 0.68%, P = 0.034) (Fig. 1; Supplement Digital Content Fig. 1). The median (25th percentile and 75th percentile) relative change in postoperative troponin I concentrations compared to baseline troponin I concentrations was 13% (− 18% to 81%) in patients in the personalized goal-directed hemodynamic management group and 65% (− 2% to 251%) in patients in the routine hemodynamic management group (P = 0.004) (Fig. 2).

**Fig. 3:**
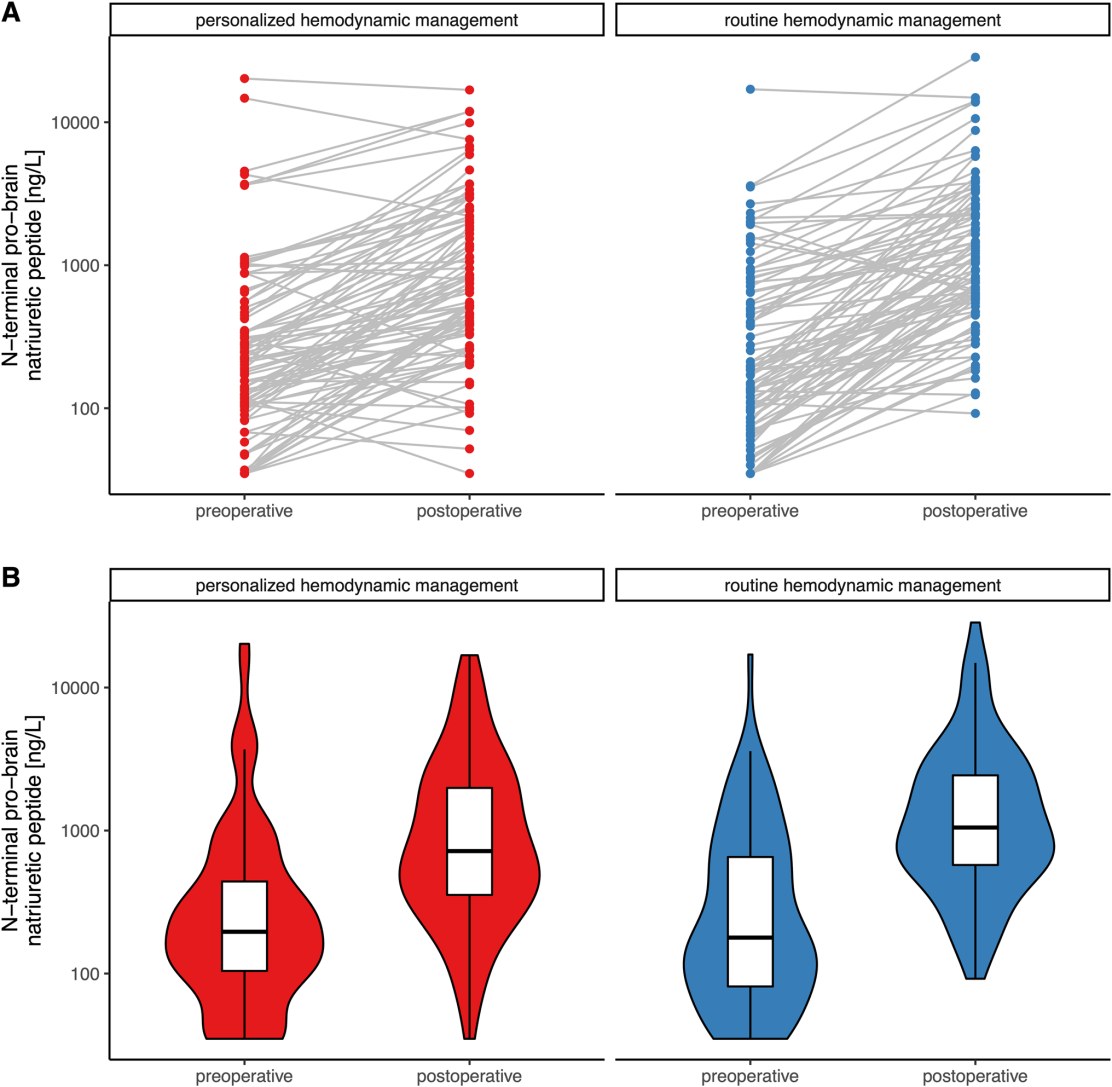
Spaghetti plots (**A**) and violin plots with overlaying boxplots (**B**) showing preoperative and postoperative N-terminal pro-brain natriuretic peptide concentrations in patients randomized to personalized goal-directed hemodynamic management and to routine hemodynamic management. Violin plots represent densities estimated by a Gaussian kernel using automated bandwidth selection with a constant area of all violins and trimming to the data range. In the boxplots, boxes represent the 25th and 75th percentile and the range between them is the interquartile range. Inside the boxes, bold horizontal lines represent medians. The whiskers (extensions from the box) indicate the lowest and highest value no further than 1.5 times the interquartile range

The median (25th percentile and 75th percentile) relative change in postoperative NT-proBNP concentrations compared to baseline NT-proBNP concentrations was 227% (96% to 664%) in patients in the personalized goal-directed hemodynamic management group and 430% (143% to 851%) in patients in the routine hemodynamic management group (P = 0.046) (Fig. 3).

## Discussion

In this post-hoc secondary analysis of a randomized clinical trial, intraoperative personalized goal-directed hemodynamic management reduced the incidence of acute myocardial injury compared to routine hemodynamic management in high-risk patients having major abdominal surgery. Additionally, personalized goal-directed hemodynamic management reduced perioperative troponin I and NT-proBNP increases compared to routine hemodynamic management.

Goal-directed hemodynamic management refers to a protocolized treatment strategy aiming to optimize global cardiovascular hemodynamics by titrating fluids, vasopressors, and inotropes to predefined hemodynamic target values [16]. On the one hand, intraoperative goal-directed hemodynamic management may improve cardiac output, oxygen delivery, and blood pressure [17]—and may thus improve myocardial perfusion and myocardial oxygen supply. On the other hand, inotropes and vasopressors may increase myocardial oxygen consumption that may result in an imbalance between cardiac oxygen consumption and oxygen supply.

The effect of intraoperative goal-directed hemodynamic management on acute myocardial injury remains scarcely investigated. In contrast to our results, perioperative goal-directed hemodynamic management—compared to usual care—did not reduce the incidence of myocardial injury and did not attenuate troponin I increases in 288 high-risk patients having major abdominal surgery in a sub-study of the OPTIMISE trial [18]. Both studies—the present one and the OPTIMISE sub-study—tested whether cardiac output-guided goal-directed hemodynamic management improves patient outcome after major abdominal surgery. Contradictory findings may be explained by different goal-directed treatment algorithms and different myocardial injury definitions.

We defined acute myocardial injury—as recommended by a consensus group [6]—according to the Fourth Universal Definition of Myocardial Infarction (2018) [7] as a relative increase in the postoperative troponin I concentration from preoperative baseline above the sex-specific 99th percentile upper reference limit; myocardial injury is considered acute if there is a rise or fall of cardiac troponin values—whether or not troponin changes are caused by myocardial ischemia [7]. This definition of myocardial injury also includes the diagnosis “myocardial infarction”—however, the diagnosis of myocardial infarction requires clinical evidence of acute myocardial ischemia (e.g., clinical symptoms, electrocardiogram changes, imaging evidence of new loss of viable myocardium or new regional wall motion abnormality) [7]. Some authors—instead of using the myocardial injury definition of the Fourth Universal Definition of Myocardial Infarction (2018) [7]—proposed the concept of “myocardial injury after noncardiac surgery” [1, 3]. Myocardial injury after noncardiac surgery is also defined by elevated cardiac troponin concentrations, but only considers elevations caused by myocardial ischemia and thus requires meticulous outcome adjudication [1, 3]. “Acute myocardial injury” and “myocardial injury after noncardiac surgery” may seem to differ only slightly—but represent two different concepts of defining postoperative myocardial injury [19].

In our study, the incidence of acute myocardial injury was 9%. The incidence of perioperative acute myocardial injury varies substantially depending on the definition [20]. In the sub-study of the OPTIMISE trial, acute myocardial injury was defined as a postoperative troponin I concentration above the 99th percentile upper reference limit and occurred in almost half of the patients [18]. In another study, the incidence of acute myocardial injury after noncardiac surgery—defined as an absolute perioperative increase in high-sensitivity troponin T of ≥ 14 ng/l (99th percentile upper reference limit: 14 ng/l)—was 16% [5]. Varying definitions of acute myocardial injury make it difficult to compare study results on its incidence [20, 21].

We not only considered perioperative troponin I changes but also investigated the effect of personalized goal-directed hemodynamic management on NT-proBNP. NT-proBNP is a biologically inactive prohormone that is released by ventricular myocytes in response to myocardial ischemia or changes in ventricular wall stretch [22]. Measuring NT-proBNP is used for diagnosis, management, and prognosis of heart failure, but in recent years it also is increasingly used for perioperative risk stratification [9, 23]. Pre- and postoperative elevated NT-proBNP concentrations in patients having noncardiac surgery are strong predictors of cardiovascular complications including myocardial injury, cardiac failure, and death [24, 25]. Postoperative NT-proBNP concentrations of ≥ 718 ng/l have been shown to independently predict 30-day mortality or nonfatal myocardial infarction in patients having noncardiac surgery [25]. In our patient cohort, personalized goal-directed hemodynamic management reduced perioperative NT-proBNP increases compared to routine hemodynamic management. Whether this translates into better patient outcome needs to be assessed in future trials.

None of the patients in the original trial developed acute myocardial infarction, i.e., elevated cardiac troponin concentrations with clinical signs of myocardial ischemia [13]. However, this secondary analysis shows that 9% of the patients developed acute myocardial injury. The clinical relevance of acute myocardial injury still needs to be determined, but it is associated with postoperative 30-day and 1-year mortality, and major adverse cardiovascular events [26]. Some recent guidelines, therefore, suggest that cardiac troponins should be routinely measured—especially in high-risk patients—before and after surgery to diagnose acute myocardial injury [27–29].

This post-hoc analysis suggests that intraoperative personalized goal-directed—compared to routine—hemodynamic management reduces the incidence of acute myocardial injury in high-risk patients having major abdominal surgery. However, this result needs to be confirmed in larger trials—that may also shed light on which hemodynamic variables should primarily be targeted to reduce acute myocardial injury. We measured postoperative troponin I and NT-proBNP just once, i.e., three days after surgery, and therefore may have missed elevated postoperative troponin I and NT-proBNP concentrations which occurred later. Nevertheless, most patients experience acute myocardial injury within two days after surgery [1].

In conclusion, in this post-hoc secondary analysis of a randomized clinical trial, intraoperative personalized goal-directed hemodynamic management reduced the incidence of acute myocardial injury compared to routine hemodynamic management in high-risk patients having major abdominal surgery. Additionally, personalized goal-directed hemodynamic management reduced perioperative troponin I and NT-proBNP increases compared to routine hemodynamic management. These findings need to be confirmed in larger prospective trials.

## Supplementary Information

Below is the link to the electronic supplementary material.

Supplementary file1 (PDF 45 kb)
