# Supplementary material for: Effect of intraoperative personalized goal-directed hemodynamic management on acute myocardial injury in high-risk patients having major abdominal surgery: a post-hoc secondary analysis of a randomized clinical trial
Source: J Clin Monit Comput. 2022 Feb 24;36(6):1775–83. doi: 10.1007/s10877-022-00826-0 (PMC9637594; doi:10.1007/s10877-022-00826-0)

Supplemental Digital Content Figure 1: Stacked bar chart showing the number of men and women with (red) and without (grey) acute myocardial injury in the personalized goal-directed hemodynamic management group and the routine hemodynamic management group.

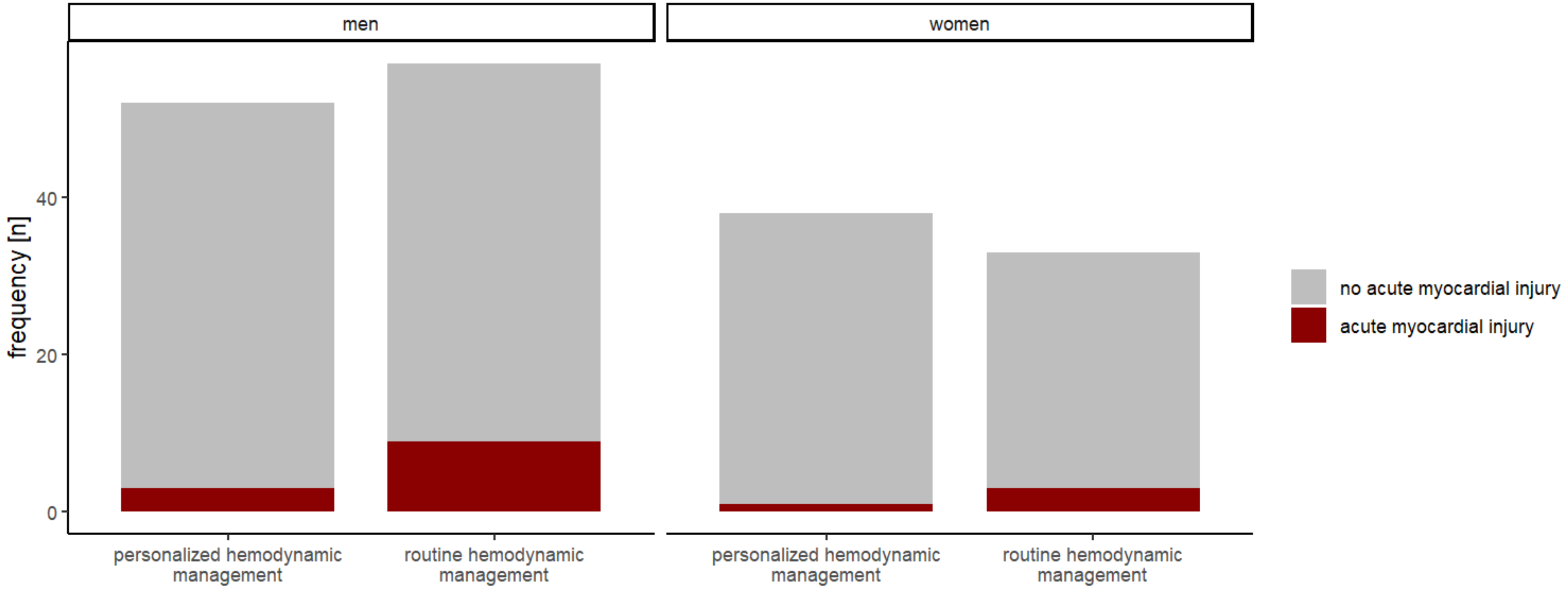

Supplement: Supplementary file 1 — Supplementary file1 (PDF 45 kb) [file 10877_2022_826_MOESM1_ESM.pdf]
